# Roles of immune cell metabolism in rheumatoid arthritis

**DOI:** 10.3389/fimmu.2026.1763130

**Published:** 2026-02-04

**Authors:** Rui Xie, Zeping Chen, Shufang Deng, Xiaofeng Jiang, Yue Feng, Wei Zhao

**Affiliations:** 1 Department of Tuina, The Third Affiliated Hospital of Chengdu University of Traditional Chinese Medicine (West District), Chengdu Pidu District Hospital of Traditional Chinese Medicine, Chengdu, China; 2 School of Acupuncture and Tuina, Chengdu University of Traditional Chinese Medicine, Chengdu, China

**Keywords:** immune cell, inflammation, metabolism, reprogramming, rheumatoid arthritis, synovial inflammation

## Abstract

Rheumatoid arthritis (RA) is a chronic autoimmune disease characterized by persistent synovial inflammation and progressive joint destruction. Recent advances reveal that immune cell metabolism plays a pivotal role in shaping RA pathogenesis. Aberrant glycolysis, lipid reprogramming, and amino acid catabolism drive functional alterations in T cells, B cells, macrophages, neutrophils, and fibroblast-like synoviocytes (FLSs), promoting inflammatory cytokine production, angiogenesis, and autoantibody generation. Key metabolites—such as lactate, succinate, and glutamine—not only serve as energy substrates but also act as immunomodulatory signals via the HIF-1α, PI3K/AKT/mTOR, and NF-κB pathways, exacerbating immune dysfunction and tissue damage. The plasticity of metabolic states contributes to Treg/Th17 imbalance, proinflammatory macrophage polarization, and FLS hyperactivation. Targeting these metabolic checkpoints has shown promise in restoring immune tolerance and alleviating disease severity. This review summarizes the complex interplay between immune cell metabolism and RA pathophysiology, highlights mechanistic insights into immunometabolic reprogramming, and discusses emerging metabolic interventions that may complement conventional RA therapies.

## 1 Introduction

Rheumatoid arthritis (RA) is a chronic autoimmune disease characterized by recurrent systemic synovitis, frequently manifesting as inflammation of the tendon sheath and synovial membrane (1). This inflammatory milieu promotes the formation of pannus—an invasive, proliferative tissue that drives joint swelling and progressive destruction of cartilage and bone (2). The pathological basis of RA involves persistent synovial inflammation with hyperplastic fibroblast-like synoviocytes (FLS) and dense infiltration of neutrophils, monocytes, and lymphocytes (3, 4). In advanced stages, sustained immune activation culminates in irreversible joint deformities and functional disability. Beyond articular damage, RA is associated with extra-articular manifestations, particularly cardiovascular complications such as ischemic and non-ischemic heart disease, and may also affect the skin, lungs, and eyes (5–7). Despite the widespread use of non-steroidal anti-inflammatory drugs, glucocorticoids, disease-modifying antirheumatic drugs, and biologics, current therapeutic regimens are limited by high cost, incomplete long-term remission, and adverse effects (8, 9).

Immunometabolism, an emerging interdisciplinary field bridging immunology and metabolism, focuses on the relationship between immune cell function and intracellular metabolic pathways, as well as their regulatory roles in disease (10, 11). Immune cells possess high plasticity and can adapt their metabolic requirements in response to inflammatory environments (12–14). Evidence suggests that immune regulation is closely linked to metabolic processes, with epigenetic mechanisms and metabolic pathways, such as glycolysis and oxidative phosphorylation (OXPHOS), playing central roles (15, 16). In RA, disruptions in glucose, lipid, and amino acid metabolism contribute substantively to immune dysregulation and disease progression (17, 18). Notably, early RA is characterized by a breakdown in T cell tolerance, where mitochondrial and lysosomal defects skew T cell differentiation trajectories (19). Rather than forming protective memory subsets, naïve T cells undergo aberrant activation, generating hyperproliferative and proinflammatory phenotypes that exacerbate synovial pathology (20, 21). Recognizing the multifactorial nature of RA, this review synthesizes current understanding of immune-metabolic crosstalk, delineating how immune cell subsets, metabolic intermediates, and regulatory signaling axes collectively drive RA pathogenesis.

## 2 Immune cells metabolic features in RA

The immune system is composed of immune organs, immune molecules, and various immune cell subsets. Key immune cell populations include T lymphocytes, B lymphocytes, neutrophils, eosinophils, basophils, monocytes, and macrophages. These immune cells play distinct and critical roles in the pathogenesis of RA (22–25).

### 2.1 T lymphocyte

T lymphocytes originate from hematopoietic stem cells in the bone marrow and subsequently migrate to the thymus, where they differentiate into CD4^+^ and CD8^+^ double-positive thymocytes (26). Following positive and negative selection, mature single-positive T cells are released into peripheral lymphoid organs and tissues to exert adaptive immune responses (27–29). During the progression of RA, T cells expand clonally in secondary lymphoid organs in response to self-antigens, leading to a breakdown of immune tolerance (30). Activated CD4^+^ T cells initiate diverse immune responses, while naïve CD4^+^ T cells in RA exhibit impaired glycolytic capacity, reduced ATP production, and increased flux through the pentose phosphate pathway (PPP) (31, 32). This metabolic shift enhances NADPH consumption and elevates intracellular reactive oxygen species (ROS) levels, thereby promoting hyperproliferation and skewing T cells toward a proinflammatory phenotype that fuels disease progression (33). In addition, regulatory T cells (Tregs) may facilitate Th17 responses under inflammatory conditions. Instability in the Treg phenotype can result in their conversion to pathogenic effector T cells that secrete proinflammatory cytokines, exacerbating autoimmune inflammation (34, 35). Moreover, the inflammatory milieu not only impairs the suppressive function of Tregs but also induces their plasticity toward Th17-like phenotypes (36).

### 2.2 B lymphocyte

B lymphocytes, originating from hematopoietic stem cells in the bone marrow, differentiate into antibody-secreting plasma cells upon antigenic stimulation, thereby orchestrating humoral immune responses (37). RA patients are typically stratified into active or remission phases, with active disease characterized by a marked reduction in circulating B cells compared to individuals in remission (38). Among B cell subsets, regulatory B cells (Bregs) exert immunosuppressive effects through the secretion of IL-10, attenuating CD4^+^ T cell proliferation and inhibiting the production of proinflammatory cytokines such as TNF-α (39, 40). Loss of this regulatory axis may perpetuate chronic inflammation in RA pathogenesis (41). Beyond their role in immune regulation, B cells are critical effectors of autoimmunity in RA, producing pathogenic autoantibodies—most notably rheumatoid factor and anti-citrullinated protein antibodies (ACPA), the latter serving as highly specific biomarkers for disease diagnosis and progression (42–44). Upon activation, B cells undergo differentiation into plasma cells that secrete these autoantibodies, thereby driving synovial inflammation and joint destruction. Evidence from experimental autoimmune arthritis models demonstrates that B cell deficiency results in diminished proteoglycan-specific immune responses and attenuated disease severity, underscoring the indispensable contribution of B cells to arthritis development (45). Moreover, B cells fulfill antigen-presenting functions, promote T cell activation, and release a repertoire of proinflammatory and costimulatory molecules (46, 47). These multifaceted activities potentiate the activation, clonal expansion, and differentiation of autoreactive T cells, thus amplifying the autoimmune cascade and perpetuating synovial pathology (38).

### 2.3 Macrophages

Macrophages, as indispensable components of the innate immune system, display extraordinary functional and phenotypic heterogeneity (48). They are pivotal not only in development and tissue homeostasis but also in immune surveillance and repair. Depending on their polarization states, macrophages adopt distinct roles—either promoting inflammation or facilitating resolution (49). Conventionally, they are classified into two major subsets: classically activated (M1) macrophages and alternatively activated (M2) macrophages, based on functional and phenotypic markers (50). M1 macrophages are characterized by robust production of proinflammatory cytokines, activation of endothelial cells, and recruitment of additional immune effectors to sites of inflammation. M2 macrophages exhibit pronounced anti-inflammatory capacity, characterized by efficient clearance of apoptotic cells and the secretion of immunosuppressive mediators (51). This functional divergence is underpinned by fundamentally distinct metabolic programs. M1 macrophages predominantly utilize glycolysis and the pentose phosphate pathway to meet their bioenergetic demands, generating minimal ATP through mitochondrial oxidative phosphorylation (52–54). This metabolic rewiring leads to a break in the tricarboxylic acid (TCA) cycle, resulting in the accumulation of immunomodulatory intermediates such as citrate, succinate, and itaconate (55). In contrast, M2 macrophages sustain intact mitochondrial respiration and rely on OXPHOS and fatty acid oxidation (FAO) to support their anti-inflammatory phenotype (52) The polarization switch from M2 to M1 macrophages entails a profound metabolic transition—from FAO and OXPHOS to aerobic glycolysis—underscoring the link between metabolism and immune function (56, 57). This metabolic plasticity underlies the emergence of pathogenic macrophage phenotypes in RA. Notably, tissue-resident synovial macrophages can form a protective barrier to preserve joint integrity and limit inflammation, whereas monocyte-derived infiltrating macrophages contribute to synovial hyperplasia, inflammatory amplification, and joint destruction (58).

### 2.4 Neutrophil and fibroblast-like synoviocyte

Neutrophils represent essential components of the innate immune system and serve as primary phagocytic effectors orchestrating host defense (59). Beyond their antimicrobial functions, they are intricately involved in immune modulation and the pathogenesis of various diseases, including RA (60). In the RA microenvironment, neutrophils predominantly rely on glycolysis and the PPP for glucose metabolism, yielding lactate as the terminal product of glycolysis to fulfill their energetic demands (61, 62). Upon inflammatory stimulation, neutrophils exert both direct and indirect effects—releasing granule-derived proteases that perpetuate chronic inflammation and tissue injury, while simultaneously engaging in bidirectional communication with other immune cells to shape the local immune microenvironment (63, 64). Neutrophils also possess immunoregulatory capacity via secretion of IL-10, a key anti-inflammatory cytokine with immunosuppressive properties (65). Deficient IL-10 production by neutrophils has been implicated in exacerbating synovial inflammation and accelerating joint destruction, underscoring their dualistic role in RA pathogenesis (66).

The synovial membrane in RA is primarily composed of two cell populations: type A synoviocytes with macrophage-like phagocytic capacity, and type B synoviocytes, or fibroblast-like synoviocytes (FLS), which are chiefly responsible for synthesizing lubricating molecules such as hyaluronic acid and lubricin to preserve joint homeostasis (3, 67). Under the inflammatory condition characteristic of RA, proinflammatory cytokines and signaling pathways stimulate aberrant activation and proliferation of FLS (68). These activated FLS not only amplify local inflammation through the release of cytokines and chemokines but also contribute to pannus formation, a hallmark of RA synovitis, and actively participate in cartilage and bone erosion (69). FLS hyperplasia contributes to a substantial increase in synovial cellularity, serving as a key link between immune cell activity and structural joint damage. FLS also mediate reciprocal interactions with immune cells that amplify chronic inflammation (70). Vascular endothelial growth factor (VEGF) is a pivotal proangiogenic mediator in RA, which can activate FLS and promote both angiogenesis and synovial hyperplasia. This feed-forward loop involving VEGF and FLS exacerbates neovascularization and joint destruction, contributing to RA progression (71–73).

## 3 The relationship between immunity and metabolism in RA

### 3.1 Influence of glycolytic enzymes on immune cell function

Glucose metabolism, particularly glycolytic flux, constitutes a fundamental determinant of immune cell function and inflammatory activation (74, 75). Glycolysis emerges as a critical metabolic program in rheumatoid arthritis, with several glycolytic enzymes exerting profound pathogenic effects. Among them, hexokinase (HK), phosphofructokinase-1 (PFK-1), and 6-phosphofructo-2-kinase/fructose-2,6-bisphosphatase 3 (PFKFB3) serve as pivotal regulatory nodes that orchestrate metabolic reprogramming in immune and stromal compartments (76–78). HK catalyzes the first rate-limiting step of glycolysis, initiating glucose catabolism. Chromatographic isolation from mammalian tissues has revealed five isoforms—HK-I to HK-IV, and the more recently characterized hexokinase domain-containing protein 1 (HKDC1)—each exhibiting tissue-specific expression and distinct subcellular localization (77, 79). HKDC1 facilitates the phosphorylation of glucose following its transport into cells, thus committing it to glycolytic metabolism (79, 80). HK mediates pathological phenotypes in RA fibroblast-like synoviocytes, including synovial hyperplasia, neoangiogenesis, pannus formation, and subsequent joint erosion (81). PFK-1 represents the second rate-limiting enzyme of glycolysis, while PFKFB3, which regulates the levels of fructose-2,6-bisphosphate (F-2,6-BP), is a potent glycolytic activator (82). Notably, PFKFB3 is markedly upregulated in the synovium, immune infiltrates, and FLS of RA patients, correlating with enhanced glycolytic throughput and aberrant cellular proliferation (83, 84). PFKFB3 enhances glycolytic flux and augments the synthesis of pro-inflammatory mediators. Inflammatory stimuli such as NF-κB have been shown to transcriptionally activate PFKFB3, thereby amplifying glycolysis (77, 83). Another key regulator, pyruvate kinase M2 (PKM2), is also elevated in RA, with increased expression observed in FLS, peripheral blood mononuclear cells (PBMCs), and inflamed synovial tissue (85, 86). Functionally, PKM2 promotes CD4^+^ T cell activation and polarization towards Th17 and Th1 subsets, fostering autoimmune inflammation. Inhibition of PKM2 has been shown to significantly reduce joint swelling and synovial inflammation in RA patients (87, 88).

### 3.2 Effects of RA metabolites on immune cells

The immune system constitutes the primary defense barrier against pathogenic invasion, yet metabolic byproducts generated during RA can profoundly modulate immune function (17). Lactate, a major metabolite of glycolysis, enhances the expression of inflammatory genes in T cells and macrophages, thereby aggravating synovial inflammation and advancing disease progression. Lactate accumulation in the RA microenvironment also promotes B cell activation and differentiation, further amplifying inflammatory responses (89). In addition, lactate facilitates Th17 cell differentiation and stabilizes Th17-associated transcription factors, accelerating the progression of autoimmune pathology (90, 91). Rheumatoid arthritis synovial fibroblasts (RASF) can utilize lactate as a metabolic substrate to sustain their proliferative and invasive behavior, leading to progressive synovial hyperplasia and joint destruction (92). Lipid metabolism is likewise dysregulated in RA, influencing the phenotype and function of macrophages (93, 94). When macrophages rely predominantly on OXPHOS, they tend to polarize toward an anti-inflammatory M2 phenotype. Conversely, in the hypoxic and inflammatory synovium of RA, M1 macrophages preferentially activate glycolysis, thereby promoting the synthesis of proinflammatory mediators and amplifying immunopathology (52, 95). Amino acid metabolism represents another critical metabolic axis in RA. Glutamine metabolism, in particular, serves as both an energy source and a biosynthetic precursor for proliferating immune cells (96, 97). In RA, heightened glutaminolysis enhances immune cell activation and effector function, contributing to persistent inflammation (18, 98). Additionally, succinate—a TCA cycle intermediate—accumulates in activated macrophages and stabilizes HIF-1α, thereby promoting VEGF production and accelerating angiogenesis and inflammation (99). Notably, succinate has been detected in the synovial fluid of RA patients, where it further activates macrophages and exacerbates disease severity (100) (**Table 1**).

**Table 1:** Immune cell metabolism and pathogenic roles in rheumatoid arthritis.

Immune cell type	Metabolic pathways	Key metabolites	Activated signaling pathways	Functional impact in RA
T Cells (Th1/Th17, Tregs)	Glycolysis, Pentose Phosphate Pathway (PPP)	Lactate, NADPH, Reactive Oxygen Species (ROS)	HIF-1α, mTOR, PI3K/AKT, NF-κB	Promotes Th17 differentiation; impairs Treg stability; induces proinflammatory cytokines (IL-17, IFN-γ)
B Cells	Glycolysis, Lactate Accumulation	Lactate	HIF-1α, PI3K/AKT/mTOR	Produces autoantibodies (RF, ACPA); supports T cell activation; releases inflammatory cytokines
Macrophages (M1/M2)	M1: Glycolysis	Succinate, Itaconate, Citrate	HIF-1α, NF-κB	M1: proinflammatory cytokine production (IL-6, TNF-α); synovial invasion
	M2: Oxidative Phosphorylation (OXPHOS), Fatty Acid Oxidation (FAO)			M2: tissue repair (suppressed in RA)
Neutrophils	Glycolysis, Pentose Phosphate Pathway	Lactate	NF-κB	Releases ROS and granule enzymes; promotes tissue damage; reduced IL-10 secretion
Fibroblast-Like Synoviocytes (FLS)	Glycolysis, Glutaminolysis	Glutamine, Lactate	HIF-1α, PI3K/AKT/mTOR, NF-κB	Drives synovial hyperplasia; promotes angiogenesis and cartilage destruction; amplifies inflammation

### 3.3 Metabolic pathways and signaling cascades affecting immune cells in RA

MicroRNAs (miRNAs), a class of non-coding RNA molecules involved in post-transcriptional gene regulation, have been implicated in RA pathogenesis. In the early stages of RA, serum levels of miR-22 are significantly elevated (101). Aberrant miRNA expression in RA affects FLSs, altering their biological function and influencing both the severity of inflammation and the immune response (102, 103). Increasing evidence suggests that miRNAs are closely associated with the onset and progression of RA. Specific miRNA expression profiles can aid not only in the diagnosis of RA but also in monitoring treatment responses (104, 105). The mammalian target of rapamycin (mTOR) is a central regulator of both innate and adaptive immune inflammatory responses. Its hyperactivation has been implicated in numerous chronic inflammatory disorders, including rheumatic diseases (106, 107). Dysregulation of mTOR signaling results in sustained inflammation and tissue injury by promoting activation of innate and adaptive immune cells and increasing the production of proinflammatory cytokines. mTOR signaling modulates the proliferation and differentiation of T and B lymphocytes, macrophages, and dendritic cells, thereby contributing to RA pathophysiology (108). In particular, the PI3K/AKT/mTOR signaling axis is abnormally activated in synovial cells of RA patients. Elevated AKT expression and PI3K-dependent activation inhibit apoptosis while promoting FLS survival and expansion, ultimately driving RA progression (109, 110). HIF-1α is a redox-sensitive transcription factor that plays a pivotal role in the inflammatory microenvironment of RA (111). HIF-1α is highly expressed in inflamed joints, where it enhances immune cell activation and promotes angiogenesis, thereby contributing to synovial hyperplasia and invasion (112, 113). Under hypoxic conditions, HIF-1α induces the expression of angiopoietin-like 4 (ANGPTL4), a lipid-regulatory adipokine that stimulates osteoclast activity and exacerbates bone erosion. ANGPTL4 is markedly upregulated in RA synovium, serum, and synovial fluid, and co-localizes with HIF-1α in a subset of osteoclasts, suggesting their coordinated involvement in disease pathology (114).

## 4 Immune cell metabolic reprogramming in RA

### 4.1 The impact of immune cell metabolic reprogramming on RA immunity

Metabolic reprogramming is a hallmark of many pathological conditions, involving pathways such as glucose, lipid, and amino acid metabolism, which ultimately alter cellular metabolic states (115, 116). Synovial tissues in RA exhibit pronounced hypoxia (117, 118). In such oxygen-deficient niches, immune and stromal cells undergo metabolic reprogramming and exhibit the Warburg effect (119). Upon antigenic activation, T lymphocytes rapidly upregulate both glycolysis and oxidative phosphorylation, fueling their proliferation and effector function (120). RA-associated metabolic reprogramming can be modulated by naturally derived compounds such as apigenin, berberine, and naringenin, which influence T cell metabolism and immune responses (54, 121). The mTOR signaling axis plays a pivotal role in RA progression by regulating T cell activation and immune function. Metabolic rewiring also suppresses proinflammatory transcription factors such as NF-κB and the JAK/STAT pathway, mitigating inflammation (108, 122).

**Figure 1:**
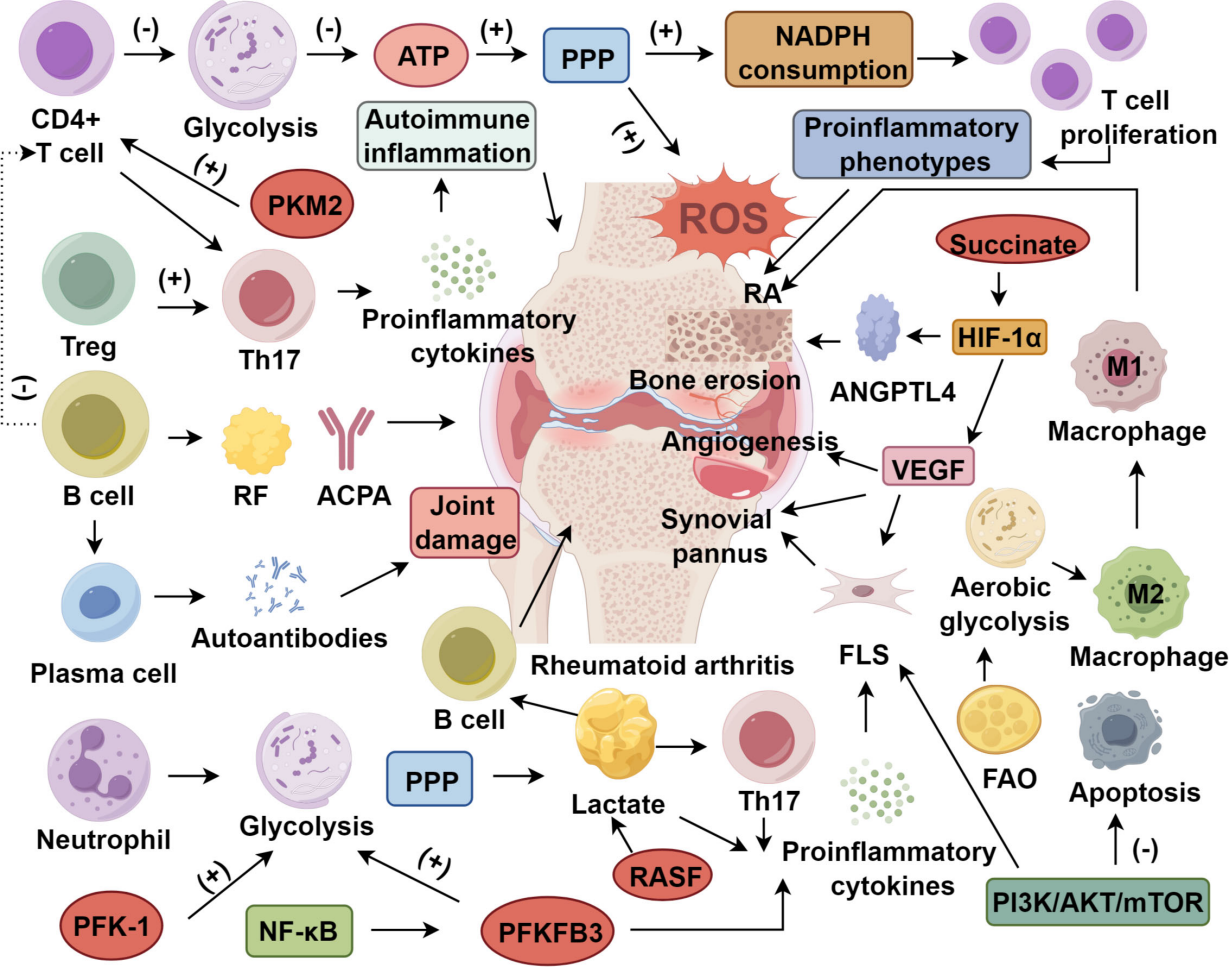
Roles of immune cell metabolism in rheumatoid arthritis. ATP, Adenosine triphosphate; PPP, Pentose phosphate pathway; FAO, Fatty acid oxidation; PFK-1/PFKFB3/PKM2, Glycolytic enzymes; RF/ACPA, Rheumatoid factor/Anti-citrullinated protein antibodies; RASF, Rheumatoid arthritis synovial fibroblasts; FLS, Fibroblast-like synoviocytes; ROS, Reactive oxygen species; HIF-1α, Hypoxia-inducible factor 1-alpha; ANGPTL4, Angiopoietin-like 4; VEGF, Vascular endothelial growth factor; NF-κB, Nuclear factor kappa-light-chain-enhancer of activated B cells; PI3K/AKT/mTOR: Phosphoinositide 3-kinase/Protein kinase B/mammalian target of rapamycin signaling axis.

During inflammation, cytokines are critical regulators. Th17 cells, in particular, are central to autoimmune processes, including those seen in RA (123, 124). RA patients frequently present with dysregulated lipid metabolism, including elevated levels of total cholesterol and high-density lipoprotein cholesterol (HDL-C), which modulate disease progression (125, 126). Lipid metabolites not only act as precursors for proinflammatory mediators but also regulate T and B lymphocyte function and differentiation, thus influencing immune homeostasis (127, 128). Metabolic reprogramming of immune cells also plays an essential role in RA-associated inflammation. Innate and adaptive immune cells exhibit metabolic signatures of increased glycolysis and reduced OXPHOS to support ATP production and biosynthesis during inflammatory activation (95, 129). Aberrations in lipid and amino acid metabolism critically influence immune cell plasticity and effector function in RA. In particular, pathways such as FAO, OXPHOS, and amino acid catabolism collectively orchestrate the immunometabolic programming of activated immune cell (130–132). Disruption of these pathways, such as impaired glutamine metabolism, can influence cytokine production and inflammation severity in RA (133) (**Figure 1**).

### 4.2 Drugs targeting immune cell metabolism in RA

A subset of naturally derived compounds has demonstrated potential in modulating immune cell metabolism in RA. Berberine has been reported to restore CD4^+^ T cell homeostasis in murine models of arthritis by downregulating miR-155 expression in both M1-polarized macrophages and CD4^+^ T cells, thereby exerting pronounced anti-arthritic effects (54). Sinomenine (SIN), a plant-derived alkaloid, was shown *in vitro* to attenuate lipopolysaccharide (LPS)-induced macrophage injury by reversing the dysregulated secretion and transcriptional expression of proinflammatory cytokines (134). Complementary *in vivo* studies confirmed that SIN effectively alleviates joint inflammation and cartilage degradation in collagen-induced arthritis (CIA) in DBA/1 mice. Additionally, total glucosides of paeony (TGP) have demonstrated immunomodulatory efficacy by upregulating anti-inflammatory cytokines, suppressing proinflammatory mediators, restoring the CD4^+^/CD8^+^ T cell ratio, and downregulating MMP-9 expression in CIA rat models, collectively contributing to the attenuation of synovial inflammation (135).

## 5 Conclusion

Metabolic reprogramming of immune cells has emerged as a central driver of RA pathogenesis, linking cellular energetics to inflammation, autoimmunity, and joint destruction. Aberrant glucose, lipid, and amino acid metabolism in T cells, B cells, macrophages, neutrophils, and fibroblast-like synoviocytes fosters pathogenic phenotypes characterized by cytokine overproduction, immune imbalance, and tissue damage. Metabolites such as lactate and succinate not only serve as bioenergetic substrates but also act as signaling mediators that activate HIF-1α, PI3K/AKT/mTOR, and NF-κB pathways, exacerbating synovial inflammation. These findings underscore the pivotal role of immunometabolism in sustaining chronic inflammatory circuits and shaping the RA microenvironment.

Targeting immune cell metabolism presents a promising therapeutic strategy to restore immune homeostasis and ameliorate disease severity. Emerging evidence highlights the potential of natural compounds—such as berberine, sinomenine, and paeoniflorin—in modulating key metabolic checkpoints and dampening inflammatory responses in preclinical RA models. Future research should focus on integrating metabolic profiling with immune phenotyping to refine patient stratification and identify novel metabolic biomarkers for disease monitoring. Additionally, the translation of immunometabolic modulators into clinical practice will require a deeper mechanistic understanding of cell-type–specific metabolic dependencies and their dynamic interplay in RA. By harnessing the therapeutic potential of immunometabolism, a new frontier may be opened in the precision treatment of autoimmune arthritis.
